# Mutations in *Neisseria gonorrhoeae* grown in sub-lethal concentrations of monocaprin do not confer resistance

**DOI:** 10.1371/journal.pone.0195453

**Published:** 2018-04-05

**Authors:** Colin P. Churchward, Alan Calder, Lori A. S. Snyder

**Affiliations:** School of Life Sciences, Pharmacy, and Chemistry, Kingston University, Kingston upon Thames, United Kingdom; Emory University School of Medicine, UNITED STATES

## Abstract

*Neisseria gonorrhoeae*, due to its short lipooligosaccharide structure, is generally more sensitive to the antimicrobial effects of some fatty acids than most other Gram negative bacteria. This supports recent development of a fatty acid-based potential treatment for gonococcal infections, particularly ophthalmia neonatorum. The *N*. *gonorrhoeae* genome contains genes for fatty acid resistance. In this study, the potential for genomic mutations that could lead to resistance to this potential new treatment were investigated. *N*. *gonorrhoeae* strain NCCP11945 was repeatedly passaged on growth media containing a sub-lethal concentration of fatty acid myristic acid and monoglyceride monocaprin. Cultures were re-sequenced and assessed for changes in minimum inhibitory concentration. Of note, monocaprin grown cultures developed a mutation in transcription factor gene *dksA*, which suppresses molecular chaperone DnaK and may be involved in the stress response. The minimum inhibitory concentration after exposure to monocaprin showed a modest two-fold change. The results of this study suggest that *N*. *gonorrhoeae* cannot readily evolve resistance that will impact treatment of ophthalmia neonatorum with monocaprin.

## Introduction

Monocaprin is a powerful fast-acting bactericidal agent against *Neisseria gonorrhoeae* [1,2]. It has recently been proposed as a candidate for treatment of gonococcal eye infections, such as ophthalmia neonatorum, where topical treatment would rapidly kill the bacteria without irritating the eye [2]. As with any antimicrobial, attention must be given to possible resistance mechanisms. There are many ways that bacteria can acquire resistance to antimicrobials and some of these can be induced in the laboratory experimentally. Growth of the bacteria on media containing a sub-lethal concentration of the antimicrobial will select for genomic mutations that confer an addition level of fitness in this environment to out-compete non-mutated cells. Previously, *N*. *gonorrhoeae* has been passaged on media containing increasing amounts of the fluoroquinolone ciprofloxacin, resulting in an isolate that had 10,000 times greater resistance than the parental isolate [3]. Mutations in the *gyrA* and *parC* genes were identified; mutations in the same genes also developed naturally in clinical isolates [4,5]. By 2006, the US Centres for Disease Control and Prevention no longer recommended fluoroquinolones for treatment of gonococcal infections [6]. Trying to predict how an organism will change genetically and / or phenotypically given a set of conditions or given a certain stimuli in the laboratory is known as experimental evolution. Bacteria are commonly studied in this way as they have short generation times which make them ideal for this method of study. The now low cost of next generation sequencing means that resequencing whole genomes following experimental evolution is feasible. This “evolve and re-sequence” strategy [7,8] can identify mutations in unexpected regions of the genome.

Although the lipopolysaccharide (LPS) of most Gram negative bacteria provides some intrinsic resistance to fatty acids, *N*. *gonorrhoeae* have short lipooligosaccharide (LOS) structures and are generally more sensitive to fatty acids [1]. *N*. *gonorrhoeae* possesses other mechanisms to protect itself from the antimicrobial action of fatty acids. It has previously been shown that isolates from men that have sex with men have reduced permeability of hydrophobic agents [9]. The FarA-FarB-MtrE efflux pump has been demonstrated to confer decreased sensitivity to certain fatty acids [10]. Transcription of *farAB* is controlled by the FarR protein and integration host factor [11,12]. Therefore, mutations in *farA*, *farB*, *mtrE*, their promoters, or any of their regulators could have an effect on the resistance profile of the bacteria.

The purpose of this study was to identify genomic mutations that resulted from passage of *N*. *gonorrhoeae* on media containing sub-lethal concentrations of the monoglyceride monocaprin and to determine any changes in the minimum inhibitory concentration (MIC). Parallel cultures grown in a sub-lethal concentration of the saturated fatty acid myristic acid (C14:0). Myristic acid was chosen as a positive control for selection of genomic mutations and increase of MIC because it has very good bacteriostatic properties against *N*. *gonorrhoeae* [2] but also has a known mechanism of resistance in the *farAB-mtrE* encoded efflux pump system [10–12]. An ocular formulation of monocaprin is a promising candidate for the treatment of ophthalmia neonatorum [2], particularly in cases of antibiotic resistant gonococcal infections, therefore understanding the potential to develop resistance and the mechanisms involved are important.

## Results and discussion

The MIC values before experimental evolution were 125 μM for myristic acid and 250 μM for monocaprin. Therefore, sub-lethal concentrations of 62.5 and 125 μM were used in the experimental evolution for myristic acid and monocaprin, respectively. The non-selective, myristic acid-, and monocaprin-containing cultures were successfully grown for a total of twenty passages each. Growth on plates containing myristic acid and monocaprin was observed to be slower than those on non-selective plates, especially the first few passages on the myristic acid-containing plates. The MICs were unchanged between the starting culture and the non-selective cultures after 20 passages (Table 1).

**Table 1:** Minimum inhibitory concentrations of the passaged *Neisseria gonorrhoeae* isolates to monocaprin and myristic acid.

Isolate	Monocaprin MIC (μM)	Myristic acid MIC (μM)
Starting culture	250	125
Non-selective passage isolate 1 (NS-1)	250	125
Non-selective passage isolate 2 (NS-2)	250	125
Myristic acid passage isolate 1 (14:0–1)	500	2000
Myristic acid passage isolate 2 (14:0–2)	500	2000
Monocaprin passage isolate 1 (MG10-1)	500	1000
Monocaprin passage isolate 2 (MG10-2)	500	1000

It is known that *N*. *gonorrhoeae* have an efflux pump-based mechanism of resistance against myristic acid [10–12]. Loss of the FarR regulator of expression of the *farAB* efflux pump genes results in an increase in the MIC for those fatty acids that are substrates of the efflux pump, including myristic acid [11]. After 20 passages, the bacteria grown on media with sub-lethal myristic acid had a sixteen-fold increase in MIC compared to the starting inoculum (Table 1). One myristic acid grown replicate (14:0–1) appeared to have a growth rate advantage over the other replicate, although the MICs were the same. The MICs of the bacteria grown on sub-lethal monocaprin increased eight-fold for myristic acid in both duplicate samples. It appears that growth in the presence of sub-lethal monocaprin has conditioned the *N*. *gonorrhoeae* for growth on myristic acid resulting in an increase in the myristic acid MIC. From these results, it is evident that resistance to myristic acid can readily develop, which is not unexpected considering previous studies [10–12].

After experimental evolution, the monocaprin MIC for the samples passaged on plates containing myristic acid or monocaprin were both 500 μM (Table 1). Unlike the eight and sixteen fold change for the myristic acid MIC, the MIC for monocaprin has simply doubled that of the starting inoculum (Table 1). This suggests that passage on sub-lethal concentrations of both myristic acid and monocaprin prepares the bacterial cells for growth on a modest increase in monocaprin. Mechanisms contributing to such an increase may be related to a general stress adaptation.

The paired sequencing reads from MicrobesNG were successfully mapped to the GenBank *N*. *gonorrhoeae* strain NCCP11945 reference genome [13]. Twelve mutations within coding sequences (CDSs) were present in all the sequenced samples in comparison to the reference sequence (Table 2). These differences are either the result of sequencing errors from the original sequencing, sequencing errors in our data, or mutations that have occurred in the *N*. *gonorrhoeae* strain NCCP11945 isolate during the few passages between it being sequenced [13] and reaching our laboratory. The original sequencing was done by Sanger sequencing with an eight-fold coverage and predicted error rate of 0.15 per 10,000 bases [13], which would equate to 33 errors in the 2,232,025 bp genome. These sequencing differences identified here were either in pseudogenes or are predicted by SNAP2 [14] not to cause a functional effect in the encoded protein.

**Table 2:** Mutations in CDSs present in all sequenced samples, but not in the reference sequence.

Locus ID	Gene product	Position	Codons	Change
NGK_RS00010	DNA polymerase III subunit beta, pseudogene	2,329	GAC → GCC	D179L, nonsynonymous
NGK_RS00195	Glutamate-1-semialdehyde 2,1-aminomutase	37,377	GCC → GGC	A290G, nonsynonymous
NGK_RS07005	Glycine dehydrogenase (decarboxylating)	1,287,872	ACG → GCG	T336A, nonsynonymous
NGK_RS07575	Ubiquinone biosynthesis regulatory protein kinase UbiB	1,395,910	AAC → AGC	N382S, Nonsynonymous
NGK_RS09065	Hypothetical protein, pseudogene	1,670,097	GGT → G-T	Frameshift
NGK_RS09355	Autotransporter, pseudogene	1,718,262	GGT → G-T	Frameshift
NGK_RS10485	DNA mismatch repair protein MutS	1,926,831	ATC → AGC	I681S, nonsynonymous
NGK_RS11075	30S ribosomal protein S10	2,031,372	TTT → TCT	F21S, nonsynonymous
NGK_RS11400	Lipid-A-disaccharide synthase	2,086,880 2,086,888	GAT → GAA ATA → AAA	D114E, nonsynonymous I117K, nonsynonymous
NGK_RS11740	Diaminopimelate decarboxylase	2,155,020 2,155,023	GAC → CTT CTG → ATC	D355L, nonsynonymous L356I, nonsynonymous

Ten unique mutations were found in CDSs of the non-selectively passage cells (Table 3), although none of these were present in both replicates (Table 3). These changes are indicative of the general rate at which mutations can occur and become part of the bacterial population. Four of the mutations are present in all of the sequencing reads from the isolate, whilst the remainder are present in the majority of reads. Each of these CDS mutations would result in a nonsynonymous change, including two that generate premature termination codons, albeit late in the gene for NGK_RS05395 at 351/400, and one that alters the initiation codon to the less favoured TTG (Table 3). These cultures have been continuously maintained on standard gonococcal media and passaged to fresh media every two to three days. This is often standard practice in research laboratories where cultures can be kept growing in incubators for extended periods of time. However as evidenced here, mutations can arise via repeated passage that may impact downstream experimental use of the bacterial culture. Care must be taken to use minimally passaged isolates from well-maintained freezer stocks particularly when comparing parent and experimentally generated mutants. When comparing results to the reference genome, the potential for mutations to have arisen in the course of experiments must be considered.

**Table 3:** CDS mutations found only in non-selectively passaged *N*. *gonorrhoeae* experimental evolution sequencing data.

Locus ID	Gene product	Position	Codons	Change	Isolate*	Reads^
NGK_RS00080	Preprotein translocase subunit SecG	14,279	CCG → CTG	A51V, nonsynonymous	NS-2	15/18
NGK_RS00215	Ribosomal protein L11 methyltransferase	39,992	CGG → CTG	G191V, nonsynonymous	NS-2	13/13
NGK_RS00290	Hypothetical protein	57,720	AAG →GAG	K86G, nonsynonymous	NS-1	15/29
NGK_RS00605	Two component sensor kinase	124,256	ATG → TTG	Start to less favourable start	NS-2	2/3
NGK_RS03680	Membrane protein	668,244	GAC → TAC	D84Y, nonsynonymous	NS-1	18/18
NGK_RS05395	Type I restriction endonuclease subunit S	978,753	CAA → TAA	Q351STOP, premature stop	NS-2	2/2
NGK_RS06935	Adenine phosphoribosyltransferase	1,273,919	GGC → GAC	G135D, nonsynonymous	NS-2	15/16
NGK_RS09755	Fimbrial protein	1,795,528 1,795,532	CAA → AAA AAG → ACG	Q90T, nonsynonymous K91T, nonsynonymous	NS-2	4/7
NGK_RS11565	Bifunctional glutamine synthetase adenylyltransferase / deadenyltransferase	2,118,245	CAA → TAA	Q364STOP, Premature stop	NS-1	34/34

* mutation identified in either the non-selective isolate 1 (NS-1) or the non-selective isolate 2 (NS-2) culture.

^ number of sequencing reads containing the mutation identified.

**Fig 1:**
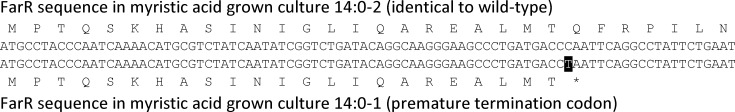
Mutations identified in *N*. *gonorrhoeae* grown with sub-lethal myristic acid. The SNP in myristic acid grown culture 14:0–1 is present at the 70^th^ nucleotide into *farR* (black highlight) and results in the formation of a premature stop codon after 23 amino acids (*). The full length FarR is 146 amino acids.

Six sequence differences were found in CDSs from the samples passaged on sub-lethal myristic acid (Table 4). A SNP mutation was identified in the 24^th^ codon of *farR* that changed it from a glutamine (Q) codon to an *ochre* stop codon in one isolate (Fig 1). This mutation is present in all sequence reads from this sample, however it is not present in any of the sequence reads of the duplicate myristic acid sample. This mutation would prevent expression of FarR, resulting in overexpression of the *farAB* encoded fatty acid efflux pump system [10–12]. This sample was observed to have a growth advantage over its other replicate but did not show a greater MIC compared to the other replicate (Table 1). The FarR transcriptional regulator also activates *glnA* responsible for glutamine biosynthesis [15] in *N*. *gonorrhoeae* and *nadA* in *N*. *meningitidis* [16]. This mutation is not surprising as mutations in the promoter region and open reading frame of another transcription regulator of an efflux pump system, *mtrR*, has been demonstrated to confer decreased susceptibility to some common antimicrobials [17, 18]. It is interesting to note that the mutation does not confer a higher level resistance to myristic acid compared to the parallel culture.

**Table 4:** CDS mutations found only in sub-lethal myristic acid passaged *N*. *gonorrhoeae* experimental evolution sequencing data.

Locus ID	Gene product	Position	Codons	Change	Isolate*	Reads^
NGK_RS00315	FarR regulatory protein	61,543	CAA → TAA	Q24STOP, premature stop	14:0–1	40/40
NGK_RS03680	Membrane protein	669,035	Deletion of 4 codons	Deletion	14:0–1	13/15
NGK_RS07715	Lysine-tRNA ligase	1,428,182	CCG → TCG	P151S, nonsynonymous	14:0–1	24/24
NGK_RS09755	Fimbrial protein	1,795,512 1,795,541	TTC → TTA TTA → TCA	F84L, nonsynonymous L94S, nonsynonymous	14:0–1 14:0–2	9/18 3/36'
NGK_RS11260	Valine-tRNA ligase	2,055,439	GTG → ATG	V187M, nonsynonymous	14:0–2	26/26

* mutation identified in either the myristic acid passaged isolate 1 (14:0–1) or the myristic acid passaged isolate 2 (14:0–2) culture.

^ number of sequencing reads containing the mutation identified.

' non-consensus.

**Fig 2:**
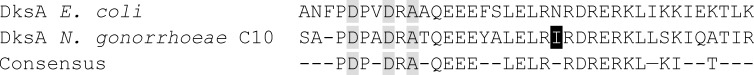
Mutations identified in *N*. *gonorrhoeae* grown with sub-lethal monocaprin. An alignment of a portion of DksA from *E*. *coli* (DksA *E*. *coli*) and the monocaprin culture mutation of *N*. *gonorrhoeae* (DksA *N*. *gonorrhoeae* C10). The grey highlighting indicates residues important in RNA polymerase binding in *E*. *coli*, conserved in *N*. *gonorrhoeae*. The black highlight indicates the mutation in *N*. *gonorrhoeae* grown with sub-lethal monocaprin.

The monocaprin passaged isolates had two non-synonymous SNP mutations (Table 5), both of which were predicted by SNAP2 to have a functional effect. The mutation in the phosphate permease protein was only present in one isolate. This is a membrane protein which is responsible for passively allowing entry of phosphate ions into the cell. It is not clear what effect this mutation would have on the cells ability to protect it from monocaprin. The mutation in *dksA* (NGK_RS106025) is present in all of the sequencing reads from both monocaprin grown cultures. The main role of DksA, a zinc-containing multi-functional protein, is as a transcription factor that binds directly to RNA polymerase and negatively regulates rRNA expression by destabilizing rRNA::promoter complexes [19], positively regulates several amino acid biosynthesis genes [19], and regulates *fis* expression [20]. An important gene regulator, DksA also inhibits transcript elongation, exonucleolytic RNA cleavage, pyrophosphorolysis, and increases intrinsic termination (http://www.uniprot.org/uniprot/P0ABS1), as well as being involved with RecN in repair of DNA double-strand breaks [21] and suppression of *dnaK* [22]. The mutation causes a substitution of the 75^th^ amino acid from a threonine to an isoleucine (Fig 2), predicted by SNAP2 to have a functional effect on the protein with a change in the predicted secondary structure. The D71, D74, or A76 residues of the *E*. *coli* homolog form part of the coiled-coil tip that is responsible for the DksA-specific effects on open complex formation [23, 24]. These amino acids are conserved in *N*. *gonorrhoeae* and the T75I change is 12 amino acids away from these conserved residues (Fig 2). The protein appears to be highly conserved within *N*. *gonorrhoeae*; at the time of writing there were 322 DksA protein sequences in the GenPept database and of these 319 (99%) have an amino acid sequence identical to *N*. *gonorrhoeae* strain NCCP11945. The remaining three have separate single amino acid substitutions, none of which were at residue 75. DksA is involved in the ‘stringent response’ which is the stress response that occurs when bacteria are exposed to heat shock, experience stress conditions, or are starved of essential cellular components such as amino acids, fatty acids, or iron [25].

**Table 5:** CDS mutations found only in sub-lethal monocaprin passaged *N*. *gonorrhoeae* experimental evolution sequencing data.

Locus ID	Gene product	Position	Codons	Change	Isolate*	Reads^
NGK_RS03680	Membrane protein	668,977	Deletion of 4 codons	Deletion and frameshift	MG10-2	26/31
NGK_RS07610	Iron complex outer membrane protein	1,402,968	AGC → AGT	S21S, synonymous	MG10-2	40/40
NGK_RS08405	Phosphate permease	1,565,103	GCC → GTC	A509V, nonsynonymous	MG10-1	16/31
NGK_RS08890	Magnesium transporter	1,635,913	CCG → CCA	P48P, synonymous	MG10-1	31/59
NGK_RS10625	RNA polymerase-binding protein DksA '	1,953,691	ACC → ATC	T75I, nonsynonymous	MG10-1 MG10-2	53/53 62/62

* mutation identified in either the monocaprin passaged isolate 1 (MG10-1) or the monocaprin passaged isolate 2 (MG10-2) culture.

^ number of sequencing reads containing the mutation identified.

' identified via pBLAST analysis.

All four samples passaged on media containing sub-lethal antimicrobials showed an increase in MIC for both antimicrobials, suggesting that mechanisms of survival in the presence of one confer cross-resistance to the other. However, no common mutations in CDSs across these four sets of sequencing data were present. The increases in MICs may therefore be a result of general adaptation to stressful growth conditions, rather than being due to specific resistance mechanisms against monocaprin or myristic acid. A modest increase in MIC such as those observed here for monocaprin (Table 1) could arise due to an adaptation of the bacterial cell to the stresses experienced by exposure to the antimicrobials, due to selection for a portion of the population expressing a different phase variable repertoire of outer membrane proteins or LOS with different permeability, or other general changes that are not specific to monocaprin resistance.

The promoters of *farR*, *farAB*, *mtrR*, and *mtrCDE* were examined for any signs of mutation in the genome sequence data. In *N*. *gonorrhoeae*, modified promoter regions can enhance the expression of efflux pumps; a mutation in the sequence upstream of *mtrC* acts as an alternative promoter region enabling transcription of *mtrCDE* without MtrR control [26]. No mutations in these regions were observed in the experimental evolution sequencing data. Of note, *N*. *gonorrhoeae* strain NCCP11945 used in this study has a single base deletion in the promoter located inverted repeat within the *mtrR mtrC* promoter region. This single base mutation results in a loss of expression of MtrR and increased expression of the MtrCDE efflux pump in *N*. *gonorrhoeae* strain FA19 [27], which suggests that overexpression of the MtrCDE efflux pump is insufficient to confer resistance to monocaprin. Further, the FarR mutant here would be overexpressing both MtrCDE and FarAB-MtrE, yet the MIC of monocaprin in this isolate (14:0–1, Table 4) remains 500 μM (Table 1).

Ophthalmia neonatorum, infection by *N*. *gonorrhoeae* of the eyes of newborns, is due to transmission of the bacteria from the infected birth canal of the mother to the eyes of the infant during birth. This is a ‘dead-end’ infection; the bacteria cannot transmit to a new host from the infant eye infection. As a result, any treatment applied to eliminate ophthalmia neonatorum that is not used to also treat reproductive tract infections is isolated to the patient, with no chance of resistant organisms evolving in the host and being transmitted to others. Monocaprin-based formulations have been shown to rapidly kill *N*. *gonorrhoeae* without causing irritation [2]. For this reason, we did not apply the strategy used previously by Belland *et al*. [3] of subjecting the bacteria to increasingly higher concentrations of monocaprin over time. For this particular clinical manifestation and this particular proposed application, given the rapid killing time of monocaprin and ‘dead-end’ nature of the infection, there is no scope for resistance to evolve within the host. Therefore, our experimental design here did not include a progressive increase in monocaprin concentration as had previous resistance studies [3]. Additional evidence presented here demonstrates that the opportunity for evolution of resistance to such a treatment is minimal within the patient and within the timeframe of treatment, therefore further supporting the development of these candidates as treatments for gonococcal eye infections.

Our results parallel those of previous researchers investigating the potential for resistance to emerge to monoglycerides. In *Staphylococcus aureus*, continuous passage on media containing sublethal concentrations of monolaurin for a year did not generate resistance [28]. It is hypothesized that resistance to monoglycerides is not seen and are less likely to develop than resistance to antibiotics because there are multiple modes of antibacterial action that would all need to be overcome [29].

In conclusion, *N*. *gonorrhoeae* strain NCCP11945 adapted to growth in media containing sub-lethal concentrations of the fatty acid myristic acid and the monoglyceride monocaprin. Passage on either of these two antimicrobials resulted in a doubling of the MIC for monocaprin, likely due to general stress adaptation. Passage on monocaprin or myristic acid resulted in eight- and sixteen-fold increases, respectively, in the myristic acid MIC. *N*. *gonorrhoeae* is known to have mechanisms of resistance to myristic acid, such as efflux pumps [10–12]. It appears that the bacteria were able to increase their ability to withstand the presence of myristic acid just by being in an environment surrounded by a similar hydrophobic agent. However, the MIC for monocaprin showed only a modest two-fold increase in MIC, suggesting that it would be a suitable candidate for treatment of gonococcal infections, such as ophthalmia neonatorum [2]. Genome sequencing revealed that the mutations from the monocaprin-containing culture are of a more general nature than those from the myristic acid cultures. It is likely that the mutations in *dksA*, present in both monocaprin cultures, have compensated for stress upon the cell due to the presence of monocaprin in the culture and that the small MIC increase observed here is the extent of what can be achieved by general adaptive mutations. The likelihood that monoglycerides such as monocaprin have multiple modes of antimicrobial action and that resistance have not emerged after a year of passage in other species [28, 29] is encouraging for their application as antimicrobials. Monocaprin is therefore a promising candidate for the treatment of gonococcal infections such as ophthalmia neonatorum.

## Methods

### Bacterial growth and experimental evolution

*N*. *gonorrhoeae* strain NCCP11945 was grown in three conditions: on standard GC agar (GC base (Oxoid) with Kellogg’s supplements [30]); on GC agar with sub-lethal monocaprin (Sigma-Aldrich; C10:0MG; 125 μM); and on GC agar with sub-lethal myristic acid (Sigma-Aldrich; C14:0; 62.5 μM). Duplicate cultures were grown for each condition. Minimally passaged *N*. *gonorrhoeae* strain NCCP11945 received from the sequencing project [13] was incubated at 37°C, 5% CO_2_ for 48 hours. Bacterial cells were removed into 1 ml GC broth to a turbidity of 0.5 McFarland standard and used to create a continuous streak over a whole GC agar plate using a sterile cotton swab. For the first passage, six GC plates were inoculated: two GC agar; two GC agar with 125 μM monocaprin; and two GC agar with 62.5 μM myristic acid. Cultures were passaged every 48–72 hours for 20 passages.

### Minimum inhibitory concentration

The minimum inhibitory concentration (MIC) was determined by agar dilution. GC agar plates were made containing 500 mM, 250 mM, 125 mM, 62.5 mM, 32.25 mM, and 16.125 mM of either myristic acid or monocaprin. Bacterial cells were suspended in 1 ml GC broth to a turbidity of 0.5 McFarland standard and used to create a continuous streak over the whole plate with a sterile cotton swab. Plates were incubated at 37°C, 5% CO_2_ for 48 hours. The lowest concentration which prevented growth was deemed the MIC. The sub-lethal concentration was the highest concentration which did not prevent growth.

### DNA extraction and genome sequencing

A Qiagen Gentra Puregene Yeast/Bac kit was used to extract DNA from 500 μl of a bacterial suspension in GC broth equivalent to a 0.5 McFarland standard. DNA from the 20^th^ passage of the six experimental evolution cultures and DNA from the starting inoculum were sent to MicrobesNG (University of Birmingham, Birmingham, UK, supported by the BBSRC, grant number BB/L024209/1) for whole genome Illumina HiSeq 2x250 bp paired-end sequencing.

### Genome sequence data quality control, assembly, and analysis

FastQ files of trimmed paired read data, were downloaded from the MicobesNG server. Read files were checked using FastQC version 0.11.2 (http://www.bioinformatics.babraham.ac.uk/projects/fastqc) and aligned using UGENE version 1.20.0 [31, 32] Bowtie2 plug-in [33] against the published sequence of *N*. *gonorrhoeae* strain NCCP11945 (accession number NC_011035.1) [13]. Assemblies in UGENE were checked manually for identified variant positions. Genes containing sequence variants were identified and predicted consequences determined using SNAP2 [14]. The promoter regions of *farR*, *farAB*, *mtrR*, and *mtrCDE* were subject to in-depth analysis.
